# Morphometrics and processing yield of *Cucumaria frondosa (Holothuroidea)* from the St. Lawrence Estuary, Canada

**DOI:** 10.1371/journal.pone.0245238

**Published:** 2021-01-22

**Authors:** Catherine M. Couillard, Domynick Maltais, Rénald Belley

**Affiliations:** Fisheries and Oceans Canada, Maurice Lamontagne Institute, Mont-Joli, Québec, Canada; Tanzania Fisheries Research Institute, UNITED REPUBLIC OF TANZANIA

## Abstract

Sea cucumber *Cucumaria frondosa* have highly variable whole body mass and length, and are usually sold to Asian markets as dried gutted body wall. Understanding the relation between size and yield of dry product is essential for resource conservation and for economic purposes. In this study, stock-specific mass and length recovery rates were estimated for *C*. *frondosa* captured by dredging or diving at various depths and seasons on the South shore of the St. Lawrence Estuary, along Gaspé Peninsula, and processed in a commercial plant. The processing yield in dry product mass per sea cucumber was more than 1.5 times larger for sea cucumbers collected at 26–47 m depth compared to those collected at 9–16 m depth. Within each strata, there was little variation in the processed body mass, seasonally or spatially. Recovery rates based on gutted mass for this stock (13.4─14.5%) varied little among depths and seasons, despite observed seasonal and bathymetric variation in reproductive status. In contrast, recovery rates based on whole body mass and length were highly variable both seasonally and spatially. Stress related to dredging or post-capture handling induced important variable body contraction and water content, leading to variation in body length, mass and shape of sea cucumbers having the same processed body mass. Gutted mass was the best metric to predict processed body mass and to estimate size whereas whole body length was the least reliable. New stock-specific information on variability of body mass, length, and recovery rates induced by capture, and on seasonal and bathymetric variation in reproductive status and processing yields will be used for the design of future stock assessment surveys, and for stock conservation.

## Introduction

**Fig 1:**
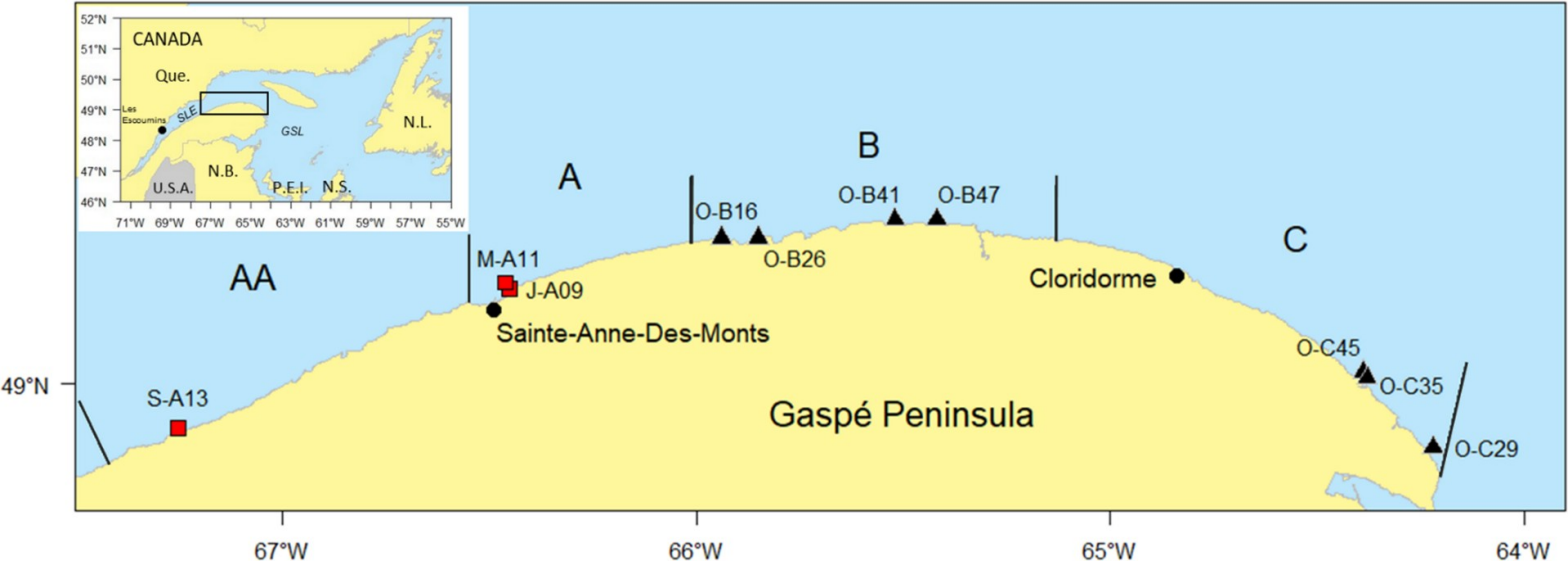
Map of sampling sites for sea cucumber (*Cucumaria frondosa*) collected on the South shore of St. Lawrence Estuary, QC, Canada. Diving (red squares) or dredging (black triangles) sites are indicated. Canadian provinces: Que., Québec, N.B., New-Brunswick, N.S., Nova Scotia, N.L., Newfoundland and Labrador, P.E.I., Prince Edward Island. SLE, St. Lawrence Estuary and GSL, Gulf of St. Lawrence. Limits of the sea cucumber fishing areas are presented (vertical black lines). Sampling group identification: M, May, J, July, S, September, O, October, Fishing area -AA, -A, -B, or -C and—water depth (last 2 numbers, m).

The orange-footed sea cucumber, *Cucumaria frondosa*, is the most abundant species of sea cucumber in the North Atlantic, and is widely distributed from Cape Cod, Massachusetts, USA, up to the Arctic Ocean, most commonly at depths from 20 to 100 m, on hard bottom (reviewed by [1, 2]). On the East coast of North America, fishing of *C*. *frondosa* began in Maine in the 1980s, and has spread to Canada in the Maritime region in the 1990s, and then to Newfoundland in the early 2000s [1, 3]. In the Province of Quebec (QC), Canada, an experimental fishery began in 2008 on the South Shore of the St. Lawrence Estuary (SLE) along Gaspé Peninsula, and in 2009, on the North Shore of the SLE near Havre-Saint-Pierre [4] (Fig 1). After capture, sea cucumbers are eviscerated, cooked, dried, and sold in the Asian market. Sea cucumbers are used for human consumption, and as a traditional medicine for the treatment of various ailments including arthritic pain, asthma and hypertension, and are a source of numerous bioactive compounds [2, 5].

The quality [6] and market value [7] of dry sea cucumber are determined by various factors including species, size, mass and the body wall thickness. For several species of sea cucumbers, mostly tropical species, recovery rates have been determined to relate fresh body mass or length at capture to dried mass and length after processing [8–10]. Recovery rates vary among species (ranging from 2 to 20%) and are also influenced by body size, processing method, level of dryness and possibly the sea cucumber origin [6, 10, 11]. Dried sea cucumber experimentally produced from *C*. *frondosa* collected in winter months of 1980–1983 at 9–11 m depth in Nova Scotia represented 5% of the whole body mass [12]. For this species, no information has been published on recovery rates after industrial processing and on their spatio-temporal variation. For resource conservation and economic considerations, it is important to maximize the income from each sea cucumber withdrawn from the stock by considering not only its size but also the dry product [13]. Whole body mass and length are highly variable in sea cucumbers since they are influenced by the quantity of seawater or food retained in the body, and the state of contraction of the body wall. Dry body mass and length after processing are less prone to variation than the whole body mass and length at capture. Availability of stock-specific recovery rates allow estimation of fresh body mass or length from processed product measurements and vice versa, providing useful information to support fisheries management decision [11]. For example, it could be used to set fishery areas boundaries and/or seasons to improve processing yields, or to obtain more accurate information on fresh size of sea cucumbers processed, to facilitate enforcement of size limits.

In this study, we estimate mass and length recovery rates for *C*. *frondosa* captured on the South shore of the SLE, along Gaspé Peninsula and processed at the Cloridorme processing plant. The variation of these recovery rates are examined in relation to sex, reproductive status, season, depth and site of capture. The capacity of different size metrics to predict the yield after processing is also assessed.

## Materials and methods

### Sample collection

On the South shore of the SLE, there are four sea cucumber fishing areas (Fig 1). In areas AA and A, sea cucumbers are collected by diving, at depths lesser than 18 m. Although dive fishing is allowed in areas B and C at the same depths, most fishing activities are performed using modified Light Green Sweep Urchin dredges at 32 to 42 m, to reduce bycatch and user conflicts. Dredges used in Quebec have a maximum width of 3.65 m [4]. A minimum size limit of 114 mm (contracted body length) has been in effect in all zones since 2013.

Commercial size sea cucumbers were collected at different seasons, in different fishing areas and at various depths to increase variability in recovery rates (S1 Table). Collection sites access of scientific teams was authorized by Fisheries and Oceans Canada through approval of detailed mission plans. Collection by the fisherman was done within the conditions of his fishing license, granted by Fisheries and Oceans Canada. Most samples, except those collected in spring and summer, were kept in flowing seawater without food for less than 24 h prior to dissection. Samples collected by diving in spring and summer were put immediately into coolers filled with aerated seawater from sampling site and ice packs, and transported alive to the laboratory. They were kept in a tank with running seawater and fed daily, 5 days a week, until 24 h before dissection with a mixture of commercially available micro-algae concentrates [14]. Samples collected by diving in September stayed in pans out of water for approximately 1.5 h before being transported to the laboratory as described above. In October, sea cucumbers were collected with the RV CCGS Leim using a dredge (2.44 m width) on wheels developed by Micmacs of Gesgapegiag (Fig 1 and S1 Table). At each site, random samples of sea cucumbers were measured (as described below). They were then put into individual mesh bags and kept for up to 24 h until dissection in seawater from the collection site.

### Dissection, biometric measurements and processing

Whole body measurements were taken on contracted individuals (S1A Fig). Contraction was induced by capture for sea cucumbers measured immediately after dredging, or by handling for individuals brought back to the laboratory after capture by diving. The whole body length (WBL) (straight line from mouth to anus) was measured using a fish measuring board (± 0.5 cm). The whole body width (WBW, right side/ left side) and height (WBH, dorsal side /ventral side) at the widest points were measured with a caliper (± 0.1 mm). The whole body mass (WBM) in air was measured (± 0.5 g). For each individual, three shape indices were calculated [15], [16] and [17]:

Ellipticity=(WBL−WBW)÷(WBL+WBW) (1)

$$Square\;root\;of\;the\;length-width\;product\;(SLW)=\sqrt{WBL\times WBW}$$ (2)

Volume=(π÷6)×(WBL×WBW×WBH) (3)

Sea cucumbers were eviscerated using a method similar to that used at the processing plant, Poissonnerie Cloridorme Inc., Cloridorme, QC. Using a scalpel, a ‘cocoon cut’ was made by cutting the aqua-pharyngeal bulb and the tentacle crown off and a 2 cm long incision was made through the anus. A plexiglass tube attached to a ShopVac® vacuum cleaner was inserted through the anterior opening to remove the remaining viscera and fluid. Gutted body mass (GBM) (± 0.1 g) and standard body length (GBL) (± 0.1 cm) were measured. Gonads were drained in a colander prior to measurement of the gonad mass (± 0.1 g). Sex was determined (presence of eggs in the female’s tubules). Residual body mass and gonadosomatic index were calculated:

Residualbodymass(RBM)=WBM−(Gonadmass+GBM) (4)

Gonadosomaticindex(GSI)=(Gonadmass÷GBM)×100 (5)

To assess the contributions of various body components to the WBM, various other indices were calculated:

Wholegonadosomaticindex=(Gonadmass÷WBM)×100 (6)

Wholebodywallindex=(GBM÷WBM)×100 (7)

Wholeresidualindex=(RBM÷WBM)×100 (8)

Two labelled tie-wraps were inserted through the posterior body walls (S1B Fig). Tagged body walls were stored in plastic bags at 4°C for a maximum of 24─48 h prior to processing. They were brought to processing plant and thrown by lots of 5 in boiling tanks, intermingled among commercial sea cucumbers. They were recovered after processing, involving a series of different drying steps. Processed body mass (PBM) and standard body length (PBL) were measured. Processed body walls were dried at 70°C until constant mass to assess water content (S1C Fig). For each individual, various recovery rates [6] were calculated:

Recoveryrateforbodylength=(PBL÷WBL)×100 (9)

Recoveryrateforwholebodymass=(PBM÷WBM)×100 (10)

Recoveryrateforguttedbodymass=(PBM÷GBM)×100 (11)

### Statistical analysis

Statistical analyses were conducted using the R environment for statistical analyses [18]. Significance level was set at 0.05 for all tests. Levene and the Shapiro-Wilk tests were used to verify the homoscedasticity and normality of data respectively. Whole, gutted and processed body mass and length were log-transformed prior to ANOVA, to meet these assumptions. For samples collected at various seasons by diving, two-ways ANOVAs were performed to assess the effects of two fixed factors, sampling time (3 levels) and sex (2 levels), and their interactions. For samples collected in October by dredging, two-ways ANOVAs were performed to assess effects of two fixed factors, sampling site (7 levels) and sex (2 levels) and their interactions on the same variables. Since recovery rates, water content and all calculated indices data did not fully meet parametric assumptions of normality and homogeneity of variance/covariance, non-parametric Kruskal Wallis (KW) tests followed by the Dunn’s multiple comparisons test were used to compared shape indices and recovery rates among groups. Spearman’s correlations coefficients between various size metrics and body mass after processing were calculated.

## Results

### Samples collected at different seasons by diving

**Fig 2:**
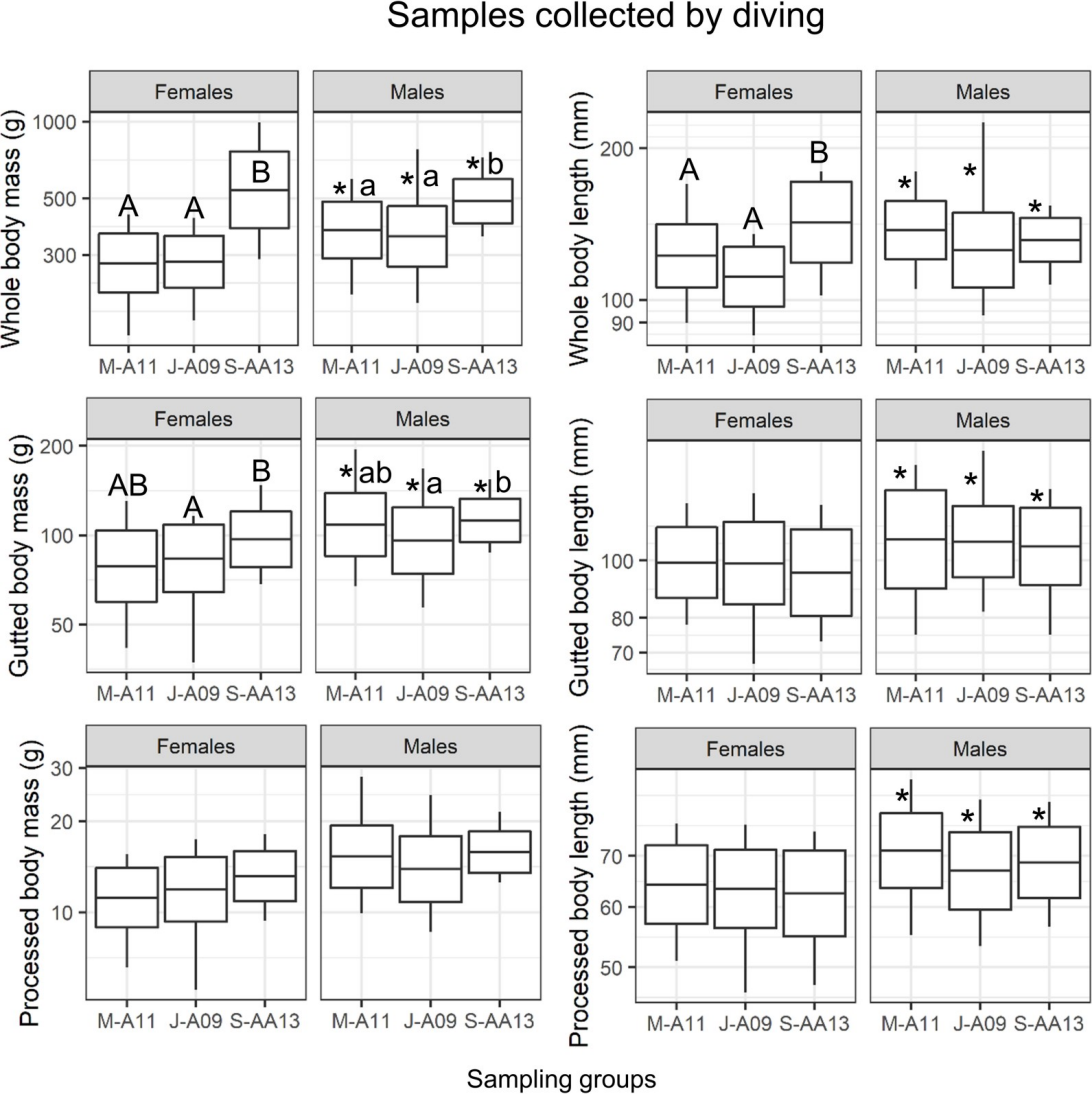
Seasonal variation in whole, gutted and processed masses and lengths in sea cucumbers collected by diving. The line in the middle of the box corresponds to the mean of the log10-transformed data, the top and bottom of the box are the mean plus and minus standard deviation and the whiskers extend outside of the box at the maximum and minimum values. Different capital and small-case letters indicate significant differences among groups within sex (ANOVA, p ≤ 0.05). Asterisks indicate significant differences between sexes. Group identification: M, May, J, July, S, September—Fisheries area -A, or -AA, and—water depth (last 2 numbers).

For whole body biometric variables, ANOVA revealed a significant interaction between sampling time and sex (S2 Table). WBM and WBL were higher in males in May and July and there was no difference between sexes in September. Therefore, the effect of time was assessed on each sex separately. Females were longer, heavier, more voluminous, had higher SLW, and lower ellipticity in September than in May or July (Fig 2 and Table 1). Similar results were obtained for males for all variables except SLW, which was lower in July and WBL, with no significant differences (Fig 2 and Table 1). For all sampling times and sexes combined (n = 151), WBL was weakly correlated to WBW (R = 0.26, p = 0.001) whereas WBW and WBH were strongly correlated (R = 0.63, p ≤ 0.0001).

**Table 1:** Variations in biometric indices for contracted body of sea cucumbers collected at different times and sites on the South shore of St Lawrence Estuary.

Group	Sex	n	ELLIP	WRI (%)	SLW	VOL (cm^3^)
Samples collected by diving						
M-A11	F	28	0.28 A (0.23–0.32)	54.6 B (50.4–60.5)	92.7 B (85.6–98.4)	273 B (223–318)
J-A09	F	24	0.25 A (0.17–0.32)	60.2 AB (54.3–63.3)	87.9 B (81.4–93.9)	248 B (220–288)
S-AA13	F	11	0.22 B (0.20–0.27)	74.0 A (69.4–78.6)	106.1 A (104–125.8)	473 A (445–597)
M-A11	M	31	0.31 A (0.24–0.35)	49.9 B (47.9–53.1)	101.7 A (94.3–107.8)	352 B (289–411)
J-A09	M	33	0.26 A (0.20–0.31)	57.1 A (62.8–67.2)	87.9 B (81.4–93.9)	248 B (220–288)
S-AA13	M	24	0.21B (0.15–0.25)	60.4 A (64.2–69.5)	104.1 A (99.2–112.4)	432 A (396–521)
Samples collected by dredging						
O-B16	F/M	9/25	0.18 bc (0.13–0.24)	63.2 bc (52.8–66.2)	90.5 b (85.8–97.5)	323 c (273–412)
O-B26	F/M	19/16	0.27 a (0.22–0.36)	68.0 a (64.2–72.8)	121 a (111–127)	685 a (501–800)
O-B41	F/M	18/17	0.27 ab (0.20–0.30)	66.6 ab (58.1–73.0)	118 a (104–125)	565 ab (390–714)
O-B47	F/M	15/20	0.27 a (0.20–0.36)	72.8 a (69.1–74.7)	119 a (108–124)	632 a (484–736)
O-C29	F/M	13/20	0.15 cd (0.07–0.22)	55.8 c (51.9–59.9)	99.2 b (94.1–107)	415 bc (365–506)
O-C35	F/M	14/19	0.08 d (0.01–0.12)	54.0 c (49.8–62.3)	97.6 b (95.1–106)	448 bc (382–538)
O-C45	F/M	21/14	0.23 ab (0.15–0.30)	72.1 a (68.9–73.8)	121 a (115–128)	698 a (604–815)

Medians (first–third quartiles) are presented for ellipticity (ELLIP), whole body residual index (WRI), square root of the length-width product (SLW), and calculated volume (VOL). Different capital and small-case letters indicate significant differences among groups for samples collected by diving or dredging respectively (Kruskal-Wallis test, p ≤ 0.05). Group identification: M, May, J, July, S, September, O, October; Fisheries areas -A, -AA, -B and -C, and water depth (last 2 numbers). Sex: F, females and M, males. Sample size (n).

Variables measured on gutted bodies also differed among sampling times, but differences were lower than those observed for whole bodies (Fig 2). Both time and sex had significant effects on GBM, without significant interaction (S2 Table). GBM was higher in September than in May and July, and higher in males than in females (Fig 2). Only sex had a significant effect on GBL, with higher values in males (S2 Table).

**Fig 3:**
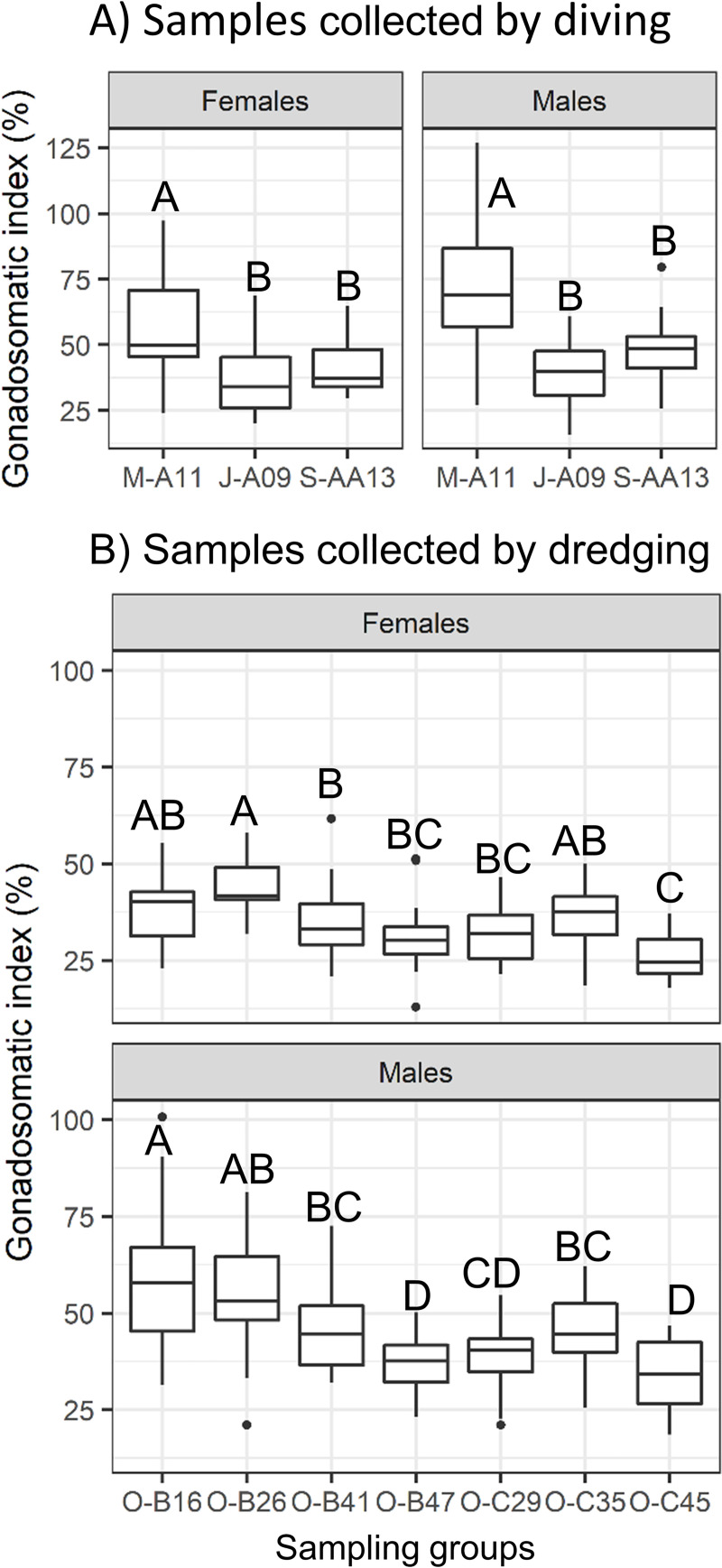
Variation in gonadosomatic indices for male and female sea cucumbers collected at different times and sites on the South shore of St. Lawrence Estuary. Results are presented for the A) diving or B) dredging studies. The line in the middle of the box corresponds to the median, the top and bottom of the box are the 75th and 25th percentiles determining the Inter Quartile Range (IQR) and the whiskers extend outside of the box at a distance of 1.5 X IQR. Outlier values are illustrated (black circles). Different capital and small-case letters indicate significant differences among groups for samples collected by diving or dredging respectively (Kruskal-Wallis test, p ≤ 0.05). Group identification: M, May, J, July, S, September, O, October -Fishing area -A, -AA, -B, or -C, and—water depth (last 2 numbers).

Gonadosomatic indices were higher in males than in females, and were higher in May than in July for both sexes (Fig 3A), Whole residual index was lower in May and higher in September (KW, p ≤ 0.0001, Table 1). In both sexes, WBM increased markedly when whole residual index reached approximately 60% (S2A Fig).

Effects of sampling time on variables measured on processed bodies was either undetectable or very weak (Fig 2). Only sex had a significant effect on PBM and PBL, with higher values in males (S2 Table). Recovery rate for whole mass was lower in September compared to May and July (KW, p = 0.0001, Table 2). Recovery rate for gutted mass was slightly lower in September than in July, with intermediate values in May (KW, p = 0.02, Table 2). Recovery rate for length did not differ among times (KW, p = 0.48). Water content of processed body walls varied from 8.1% in May, to 9.8% in September (KW, p ≤ 0.0001) (Table 2).

**Table 2:** Variations in recovery rates for sea cucumbers sampled at different times and sites on the South shore of St. Lawrence Estuary.

GROUP	RRW (%)	RRG (%)	RRL (%)	PWC (%)
Samples collected by diving				
M-A11	4.0 A (3.6–4.7)	14.1 AB (13.6–14.8)	52.3 A (47.9–57.0)	9.8 A (9.5–10)
J-A09	4.0 A (3.8–4.4)	14.5 A (14.0–14.9)	54.6 A (51.1–61.5)	9.2 B (8.9–9.4)
S-AA13	3.1 B (2.6–3.5)	14.0 B (13.5–14.4)	50.3 A (47.0–53.7)	8.1 C (8.0–8.3)
Samples collected by dredging				
O-B16	3.6 abc (3.0–4.0)	13.4 ab (12.7–14.3)	67.0 ab (57.9–70.9)	8.6 b (8.4–8.8)
O-B26	3.1 cd (2.6–3.3)	13.9 ab (13.3–14.6)	50.7 cd (46.2–55.6)	9.0 ab (8.3–9.5)
O-B41	3.3 bcd (2.6–3.8)	13.8 ab (13.3–14.4)	51.7 bc (47.1–58.5)	8.7 ab (8.3–9.9)
O-B47	2.8 d (2.5–3.2)	13.9 a (13.6–14.6)	46.0 d (42.8–49.7)	9.3 a (8.9–10.7)
O-C29	4.5 a (4.0–5.0)	13.8 b (13.4–14.2)	69.3 a (60.4–9.9)	8.8 b (8.4–9.0)
O-C35	4.5 ab (3.9–5.0)	13.9 ab (13.5–14.6)	73.9 a (68.5–83.3)	9.0 ab (8.5–9.8)
O-C45	2.5 cd (1.9–2.8)	12.9 b (12.5–13.5)	49.9 cd (47.3–56.8)	9.2 ab (8.7–9.5)

Medians (first–third quartiles) are presented for three recovery rates based on whole body mass (RRW), on gutted body mass (RRG) and on body length (RRL) and for the processed body wall water content (PWC). Different capital and small-case letters indicate significant differences among groups for samples collected by diving or dredging respectively (Kruskal-Wallis test, p ≤ 0.05). Group identification: M, May, J, July, S, September, O, October; Fisheries areas -A, -AA, -B and -C, and water depth (last 2 numbers).

GBM and WBM minus RBM were the variables most strongly correlated to PBM, with little variation of correlation strength among sampling times (Table 3A). WBM (un-corrected for residual mass), Volume, and SLW were highly correlated to PBM (R ≥ 0.75) in May and July, but not in September (R ≤ 0.35) (Table 3A). WBL was the variable most weakly correlated to the PBM.

**Table 3:** Correlations between various size metrics and body mass after processing for sea cucumbers collected at different times and sites on the South shore of St. Lawrence Estuary.

A) Samples collected by diving									
**GROUP**	**WBL**	**WBW**	**WBH**	**SLW**	**VOL**	**WBM**	**(WBM-RBM)**	**GBM**	**n**
M-A11	0.52	**0.67**	0.50	**0.78**	**0.82**	**0.82**	**0.89**	**0.98**	59
J-A09	0.47	**0.66**	0.44	**0.75**	**0.75**	**0.80**	**0.93**	**0.98**	57
S-AA13	*0*.*28*	*0*.*32*	*0*.*05*	*0*.*35*	*0*.*27*	*0*.*23*	**0.90**	**0.96**	35
Mean	0.43	0.55	0.33	0.62	0.61	0.62	0.91	0.97	
CV	29.4	36.8	74.9	38.6	49.0	53.7	2.3	1.1	
B) Samples collected by dredging									
**GROUP**	**WBL**	**WBW**	**WBH**	**SLW**	**VOL**	**WBM**	**(WBM-RBM)**	**GBM**	**n**
O-B16	*0*.*27*	**0.76**	**0.69**	**0.64**	**0.71**	**0.69**	**0.91**	**0.98**	34
O-B26	0.57	**0.78**	**0.76**	**0.81**	**0.82**	**0.86**	**0.96**	**0.98**	35
O-B41	*0*.*18*	**0.66**	0.39	0.50	0.53	0.55	**0.92**	**0.96**	35
O-B47	0.44	*0*.*32*	*0*.*34*	0.59	0.57	**0.63**	**0.94**	**0.98**	35
O-C29	*0*.*35*	0.73	**0.66**	**0.74**	**0.79**	**0.93**	**0.93**	**0.96**	33
O-C35	*0*.*20*	0.37	0.52	0.43	0.50	0.62	**0.86**	**0.96**	33
O-C45	0.36	0.53	0.46	0.65	0.68	0.72	**0.91**	**0.95**	35
Mean	0.34	0.59	0.55	0.61	0.65	0.69	0.92	0.97	
CV	40.1	32.0	29.4	19.6	18.4	15.5	3.5	1.4	

Correlation coefficients (r_s_) are presented for the whole body length (WBL), width (WBW), height (WBH) and mass (WBM), WBM minus residual mass (RBM), square root of the length-width product (SLW), volume (VOL) and gutted body mass (GBM). The strongest correlation coefficients (≥ 0.60) are in bold characters and the weakest and non-significant coefficients are in italic (p ≤ 0.05). The mean and coefficient of variations (CV) of correlation coefficients for the different groups are also presented. Sample size (n).

### Samples collected at different sites by dredging

**Fig 4:**
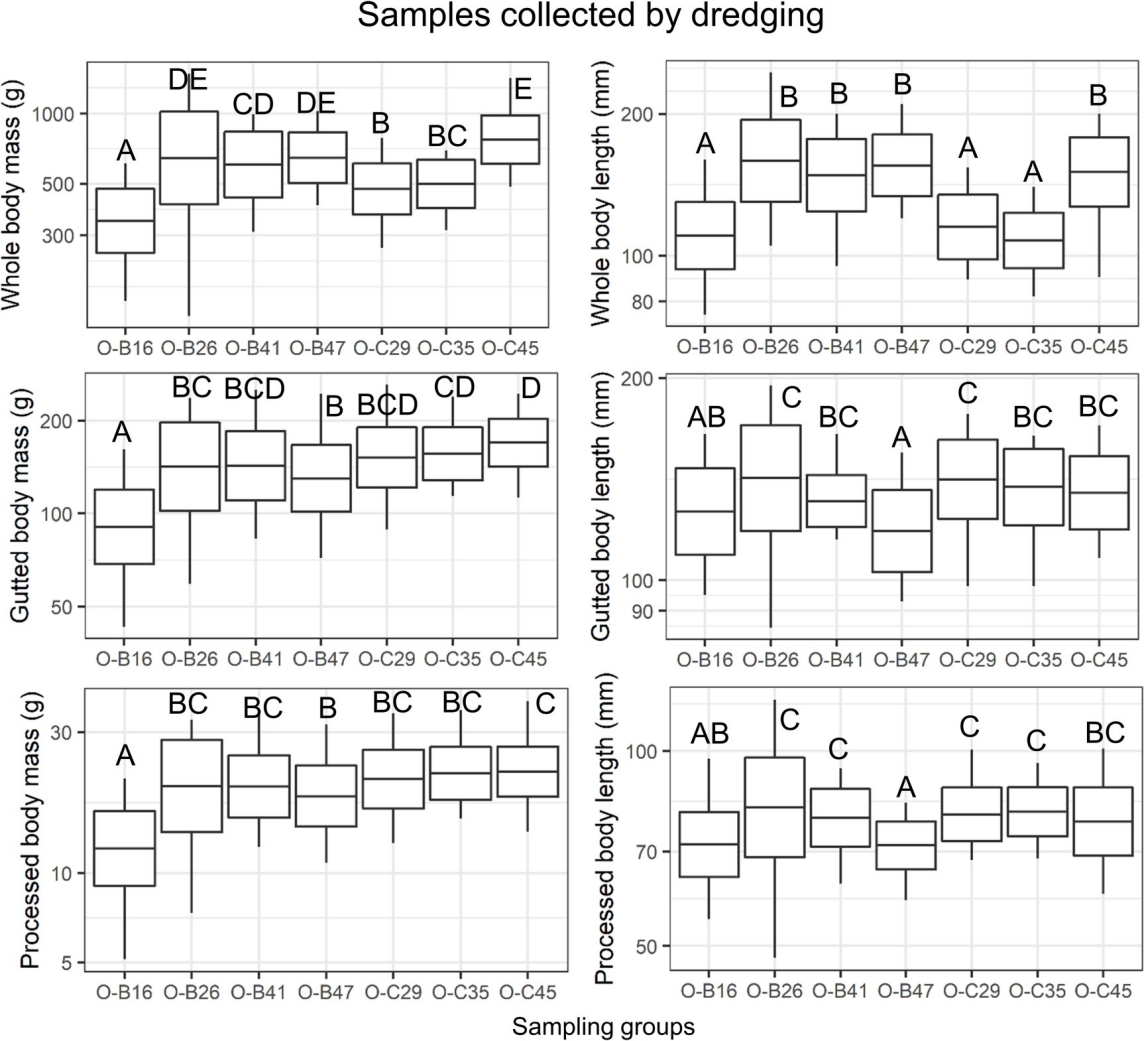
Spatial variation in whole, gutted and processed masses and lengths in sea cucumbers collected by dredging. The line in the middle of the box corresponds to the mean of the log10-transformed data, the top and bottom of the box are the mean plus and minus standard deviation and the whiskers extend outside of the box at the maximum and minimum values. Different letters indicate significant differences among groups (ANOVA, p ≤ 0.05). Group identification: O, October,—Fishing area -B, or -C, and—water depth (last 2 numbers).

There was a significant effect of sampling sites on all whole body biometric variables, with no significant effect of sex or interaction (S3 Table). Sea cucumbers captured at the shallowest water depth had the lowest body size (WBW, WBH, SLW, Volume) and body mass (WBM) compared to sea cucumbers sampled at ≥ 26 m (Table 1 and Fig 4). The same spatial pattern of variation was observed for WBL except for sea cucumber captured at O-C29 and O-C35 which stand out with a lower WBL, ELLIP, SLW and Volume compared to those sampled in the same depth range (Table 1 and Fig 4). Sea cucumbers captured at O-C45 were the widest, most voluminous, and heaviest (Table 1 and Fig 4). For all sites and sexes combined (n = 240), WBW was poorly correlated to WBL (R = 0.21) but strongly correlated to WBH (R = 0.88).

Site had a significant effect on gutted and processed body mass and length, with no significant effect of sex or interaction (S3 Table). Analyses of these metrics also indicated that sea cucumbers captured at the shallowest depth had the smallest size (Fig 4). Gutted and processed mass and length of those captured at ≥ 26 m were all in the same range except at 47 m depth where gutted length was lower (Fig 4). For both sexes, gonadosomatic indices significantly differed among sites (KW, p ≤ 0.001, Fig 3B). Gonadosomatic indices were inversely related to depth both in males (R = -0.86, p = 0.01, n = 7) and in females (R = -0.78, p = 0.04, n = 7).

In fishing areas B and C, WBM increased markedly when whole body residual index reached approximately 60% (S2B Fig). Whole body residual index was lowest for sea cucumbers captured at O-C35 and O-C29 and largest for sea cucumbers captured at ≥ 45 m (KW, p < 0.0001, Table 1). At the opposite, recovery rates based on whole body mass were highest at O-C35 and O-C49 and lowest at ≥ 45 m (KW, p < 0.0001, Table 2). Recovery rates based on gutted mass and processed body wall water content showed little spatial variation (KW, p < 0.0005, Table 2).

GBM and WBM minus RBM were the variables most strongly correlated to PBM little variation of correlation strength among groups (Table 3B). Correlations between WBW, WBH, SLW, Volume, WBM and PBM were lower and highly variable among groups, with higher values at ≤ 29 m (Table 3B). WBL was the variable most weakly correlated to PBM.

## Discussion

### Stock-specific recovery rates for *Cucumaria frondosa*

Our study provides stock-specific recovery rates for *C*. *frondosa* collected in the St. Lawrence Estuary and processed in a commercial processing plant. Observed recovery rates for whole mass (2.8–4.5%, n = 391, mean WBM = 515 g, 9–47 m depth) are lower than those reported for *C*. *frondosa* captured by dredging during winter of 1980–1983 in St. Mary's Bay, Nova Scotia, Canada, and processed in a laboratory (5.0%, n = 50, mean WBM = 276 g, 5–11 m depth) [12]. They are at the lower end of those reported for tropical sea cucumbers species (3–18.6%), reviewed by Ram *et al*. [11]. In contrast, recovery rates for length (46.0–73.9%) are at the upper end of those reported for 17 tropical sea cucumber species (32.6–55.0%) [11]. Compared to most tropical species, the dry product for *C*. *frondosa* is smaller in size [2]. In our study, median body length of *C*. *frondosa* after processing was 7.3 cm whereas mean processed body length ranged from 6.1 to 19.7 cm in other species, the majority (11 out of 13 species) being above 9 cm [9, 11].

Our estimations of GBM-based recovery rates for *C*. *frondosa* (13.4–14.5%) are slightly higher than previously published values (11.5%) for the same species, collected in Nova Scotia [12], even though their laboratory-produced dry product had higher water content (12–18%) than our commercially processed product (8–10%). One factor that could explain these lower values is winter sampling, when sea cucumbers were not feeding but using their body reserves [19]. GBM-based recovery rates for *C*. *frondosa* are at the upper end of those reported for other species (3.3–20.6%) [7, 9, 20, 21].

As previously reported for other sea cucumber species [8], recovery rates based on WBM were much more variable than those based on GBM. Residual mass, largely constituted of seawater retained in various body compartments, was a predominant source of variation. Previous studies have described a suite of behavioral changes leading to body bloating in *C*. *frondosa* exposed to various stressors. Water content in Polian vesicles and respiratory tree represented 31.0% of WBM in non-stressed *C*. *frondosa* (332 g mean WBM). and up to 78.0% in response to severe stressors such as high turbidity or low salinity [22]. This Active Buoyancy Adjustment is a common behavioral response to environmental stressors in echinoderms that enable them to increase their buoyancy and to move to locations that are more favorable [23].

### Variation associated with capture or handling

In the North Atlantic and in the Barents Sea, *C*. *frondosa* are mostly harvested using trawling and dredging whereas most other sea cucumber species are harvested by hand or diving [24]. In Canada, scientific stock assessment studies collecting data on *C*. *frondosa* biomass, density, and size distribution also rely on samples collected by dredging. Our study is the first to make the link between on-board measurements and after-processing measurements of *C*. *frondosa*, with traceability to the tow/station of origin.

Dried body mass is considered the most reliable size index in this plastic species [25, 26]. In our study, PBM, with minor variations in water content, was used as a dry mass surrogate to estimate ‘real’ body size. Except for sea cucumbers captured at 16 m depth, sea cucumbers captured at different sites had similar PBM. However, their whole body mass, length and shape varied greatly among tows. Sea cucumber have neurally-controlled mutable collagenous tissue in their body wall enabling them to modify their stiffness and body wall shape in response to predators or mechanical stimulations [26–30]. Active Buoyancy Adjustment can also induce changes in body water content, size and shape in response to stress. Potential stresses induced by dredging include crowding in nets, abrasions, concussions, changes in pressure, contact with predatory species, changes in temperature or salinity, and emersion, as shown for other benthic invertebrates (see [31], for crustaceans). Variation in abiotic conditions among hauls such as seafloor types, surface water temperature, total catch mass, wave height, and air temperature influence the survival of bycatch fish species [32]. These factors could lead to the observed variation in body dimensions among tows. In *C*. *frondosa* collected by diving, higher post-capture handling stress with a longer emersion period after capture, followed by a shorter recovery period, likely contributed to higher WBM, WBL and whole body residual index in September compared to May and July.

### Variation associated with reproduction and depth

Seasonal changes in reproductive status were not associated to changes in GBM or PBM in *C*. *frondosa* collected on the South shore of the SLE. Similarly, Hamel and Mercier [33] reported no seasonal changes in body wall mass for *C*. *frondosa* collected in 1992–1993 at Les Escoumins, on the North shore of the SLE. The reproductive cycle of this population followed a seasonal feeding cycle, with high feeding activity associated to a single phytoplankton bloom in spring followed by very low feeding activity from October to early spring [33, 34]. After spawning in May–June, the recovery phase lasted until December when gonadal growth begun, with a significant increase in GSI in March to reach a peak in May [33]. A similar cycle for feeding and reproductive activity was reported for *C*. *frondosa* in the Bay of Fundy, NB, Canada [19] and in Newfoundland [35]. In our study, the marked reduction in GSI of sea cucumbers collected in July compared to those collected at the end of May is consistent with a May–June spawning event, followed by a summer recovery period. However, sea cucumbers sampled at similar depth in October, had very high GSI, close to those observed in May and indicating a significant gonad growth. This could be the consequence of a prolonged feeding season, with a second phytoplankton bloom in August–September in addition to the spring bloom. This temporal pattern of phytoplankton blooms has been documented at an upstream monitoring station, at Rimouski, on the South shore of the SLE [36, 37]. Large-scale variation across latitudinal gradients in the timing and patterns of reproduction occur in *C*. *frondosa* [38].

In both sexes in October, GSI decreased with increasing depth, without associated variation in GBM or PBM. Differences in food supply or in temperature regimes are factors that can lead to asynchronic gonad development and/or variation in reproductive investment in echinoderms among depth strata [39–41]. *C*. *frondosa* found at depths > 850 m on the continental slope surrounding Newfoundland, Canada had markedly reduced GSI and thinner body walls compared to individuals that were sampled at < 20 m, suggesting limited food supplies [41]. Singh *et al*. [42] also reported reduction in gonadal investment in *C*. *frondosa* sampled at 80–100 m compared to individuals sampled at 10 m in Parramaquoddy Bay, NB, Canada. In contrast, Hamel and Mercier [43] observed slightly lower GSI and synchronous gametogenic cycles in *C*. *frondosa* captured at 10 m, compared to individuals captured at 110 m on the North shore of the SLE, QC, Canada. Assessment of relative contribution of sea cucumbers from different depth strata or different habitats to reproduction is needed for conservation plans.

Our results are consistent with observations reported in Campagna et al. [44] which covered the same study area during fall 2004, indicating maximal WBM at depth around 40 m and minimal at 10–20 m. Our study confirms the occurrence of truly smaller-sized individuals (based on PBM) at 9 to 16 m depth than at 26–47 m depth. Based on underwater body length measurements by divers, Hamel and Mercier [45] also reported a bathymetric size gradient, with smallest individuals at 0–20 m depth and largest at 40–60 m on the North shore of the SLE. These authors observed migration of 60–130 mm long individuals from the 0–20 m to the 20–40 m strata in the early fall. In our study, on the South shore of the SLE in October, there was no evidence for such a migration pattern since there was a clear distinction of sizes (PBM) between shallow and deeper waters. Further studies are needed to assess if a migration occurs later in the fall or in winter, as reported by Jordan [46] for *C*. *frondosa* at Lamoine Beach in Maine, USA.

### Best biometric variables to predict processed body mass

Gutted body mass was the most reliable metric to predict PBM in *C*. *frondosa*, as reported previously [26, 35, 47]. The main drawback to this metric is that it precludes returning the animal alive to the sea after measurements. A reliable alternative measurement on live specimens is immersed mass. However, it is time consuming and difficult to apply on a boat [25, 26].

Our results are consistent with previous studies indicating that body width measurement in addition to body length and calculation of size indices provide more reliable size estimation than using body length alone [16, 17, 25, 46, 48]. Calculated volume was a better predictor of WBM and PBM than SLW. Even if these indices are better body size estimators than WBL, they are still greatly influenced by the amount of water accumulated in the body, which itself is influenced by handling and capture conditions. These indices would be more easily applicable for sequential measurements of live individuals in experimental work or aquaculture, when size of organisms is relatively uniform and handling methods are standardized (as for spring and summer samples in the diving study) [49]. Our data show that these methods are less reliable (lower correlation with PBM) in field studies, when capture and handling conditions are an important source of variation in shape and size.

The apparent variation in WBM at 20–50 m was attributable to changes in residual mass (mostly water) but not to true size differences. Even though sea cucumbers were larger–sized (based on PBM), whole body length of individuals collected at 29 and 35 m depth was similar to that of individuals collected at 16 m depth, as a result of more severe bloating and body contraction during capture. Consequently, size distributions based on contracted body length of *C*. *frondosa* captured by dredging are not a reliable tool to describe the stock size-structure. In Quebec, Canada, the current protocol for scientific surveys involves counting all individuals captured in a tow and measuring contracted body length and total biomass of a representative subsample (n = 100–150). Based on our results, we suggest to add measurement of body width for calculation of volume and estimation of individual body mass, and to measure individual whole and gutted body mass in randomly selected individuals (≥ 30) from each tow. In this study, we focused on sea cucumbers of commercial size (> 114 mm, WBL) located in or nearby fishing areas. Studies on the whole size range and population distribution area are needed for further development and validation of measurement methods for different purposes, including evaluation of size-at-maturity and size distribution.

### Summary and future directions

For *C*. *frondosa* collected along Gaspé Peninsula, the main source of variation in PBM was depth, with a dry product more than 1.5-times heavier produced from the 26─47 m depth strata compared to the 9–16 m strata. Within each of these strata, PBM produced for commercial-sized sea cucumber was uniform, with little effect of local habitat conditions, seasonal or bathymetric variation in reproductive status. Thus, management decisions concerning fishing depth would likely have more impacts on processing yield per individual sea cucumber than decisions concerning fishing seasons.

Our observations of seasonal variation in the GSI at 9–13 m depth in fishing area A are consistent with a May–June spawning event. Fishing restrictions during this period would minimize risk of interference with reproduction. However, considering that we have observed a bathymetric gradient in gonad growth in October, further studies are needed to assess if there is also a bathymetric gradient in the spawning season.

Body length was a poor indicator of body size in both diving and dredging studies. These results do not support the use of a size limit based on contracted body length-at-maturity to protect the reproductive potential, unless it is very conservative and set at a size well above size-at-maturity, as recommended previously [15]. A size limit based on dry, gutted or drained body mass-at maturity would be more reliable, as suggested for the sea cucumber *Athyonidium chilensis* [50]. If these measurements are not feasible, a promising alternative could be fishing restrictions based on depth and season, providing that stock-specific bathymetric and seasonal distribution of early maturing individuals are documented.

## Supporting information

S1 Fig Sea cucumber, *Cucumaria frondosa*, at different processing stages. A) whole contracted body, B) gutted and labelled body wall, C) processed and labelled body wall.
(TIF)

S2 Fig Relation between whole body mass and whole residual index for sea cucumbers collected by diving or dredging on the South shore of St. Lawrence Estuary. A) Samples collected by diving, B) Samples collected by dredging. Results are presented for the A) diving for females and males, or B) dredging studies, for both sexes combined. Group identification: M, May, J, July, S, September, O, October,—Fishing area -A, -AA, -B or -C, and–water depth (last 2 numbers).
(TIF)

S1 Table Sampling locations, depths and time of *Cucumaria frondosa* in 2018 on the South shore of the St. Lawrence Estuary, QC, Canada. (DOCX)

S2 Table Two-way ANOVA table with sampling time and sex as main factors for whole, gutted, and processed body mass and length. Samples were collected by diving.
(DOCX)

S3 Table Two-way ANOVA table with sampling site and sex as main factors for whole, gutted, and processed body mass and length. Samples were collected by dredging.
(DOCX)

S1 Data (XLSX)

## References

[1] Hamel J-F, Mercier A. Population status, fisheries and trade of sea cucumbers in temperate areas of the Northern Hemisphere In: Toral-Granda V, Lovatelli A, Vasconcellos M, editors. Sea cucumbers. A global review of fisheries and trade. FAO Fisheries and Aquaculture Technical Paper No. 516, Rome: FAO; 2008 pp. 257–291.

[2] Nelson EJ, MacDonald BA, Robinson SM. A review of the Northern sea cucumber *Cucumaria frondosa* (Gunnerus, 1767) as a potential aquaculture species. Rev. Fish. Sci. 2012; 20: 212–219.

[3] Hamel J.-F, Mercier A. Precautionary management of *Cucumaria frondosa* in Newfoundland and Labrador, Canada In: Toral-Granda V, Lovatelli A, Vasconcellos M, editors. Sea cucumbers. A global review of fisheries and trade. FAO Fisheries and Aquaculture Technical Paper No. 516, Rome: FAO; 2008 pp. 293–306.

[4] DFO. Assessment of the Sea Cucumber fishery in Quebec’s inshore waters in 2016. DFO Can. Sci. Advis. Sec. Sci. Advis. Rep. 2017; 2017/050.

[5] Xu C, Zhang R, Wen Z. Bioactive compounds and biological functions of sea cucumbers as potential functional foods. J. Function. Foods 2018; 49: 73–84.

[6] Hair CA, Ram R, Southgate PC. Is there a difference between bêche-de-mer processed from ocean-cultured and wild-harvested sandfish (*Holothuria scabra*)?. Aquac. 2018; 483: 63–68.

[7] Purcell SW. Value, market preferences and trade of beche-de-mer from Pacific Island sea cucumbers. PloS one 2014; 9: e95075 10.1371/journal.pone.0095075 24736374 PMC3988149

[8] Skewes T, Smith L, Dennis D, Rawlinson N, Donovan A, Ellis N. Conversion ratios for commercial beche-de-mer species in Torres Strait. Australian Fisheries Management Authority, Torres Strait Research Program, AFMA Project Number: R02/1195; 2004.

[9] Purcell SW, Gossuin H, Agudo NS. Changes in weight and length of sea cucumbers during conversion to processed beche-de-mer: filling gaps for some exploited tropical species. SPC Beche-de-mer Inform Bull. 2009; 29: 3–6.

[10] Ngaluafe P, Lee J, Rodríguez Forero A, Vergara Hernández W, Agudelo Martínez V, Rougier A, et al Change in weight of sea cucumbers during processing: Ten common commercial species in Tonga. SPC Beche-de-mer Inform Bull. 2013; 33: 3–8.

[11] Ram R, Chand RV, Zeng C, Southgate PC. Recovery rates for eight commercial sea cucumber species from the Fiji Islands. Reg Stud Mar Sci. 2016; 8: 59–64.

[12] Ke PJ, Smith-Lall B, Hirtle RW, Kramer DE. Technical studies on resource utilization of Atlantic sea cucumber (*Cucumaria frondosa*). Can Inst F Sci Tec J. 1987; 20: 4–8.

[13] Purcell SW. 2010 Managing sea cucumber fisheries with an ecosystem approach Lovatelli A, Vasconcellos M, Yimin Y, editors. FAO Fisheries and Aquaculture Technical Paper. No. 520. Rome: FAO; 2010.

[14] Gianasi BL, Parrish CC, Hamel JF, Mercier A. Influence of diet on growth, reproduction and lipid and fatty acid composition in the sea cucumber *Cucumaria frondosa*. Aquac Res. 2017; 48: 3413–3432.

[15] Tuset VM, Lozano IJ, González JA, Pertusa JF, García‐Díaz MM. Shape indices to identify regional differences in otolith morphology of comber, *Serranus cabrilla* (L., 1758). J Appl Ichthyol. 2003; 19: 88–93.

[16] Yamana Y, Hamano T. New size measurement for the Japanese sea cucumber *Apostichopus japonicus* (Stichopodidae) estimated from the body length and body breadth. Fish Sci. 2006; 72: 585–589.

[17] Lee D, Kim S, Park M, Yang Y. Weight estimation of the sea cucumber (*Stichopus japonicas*) using vision-based volume measurement. J Electr Eng Technol. 2014; 9: 2154–2161.

[18] R Core Team. R: A language and environment for statistical computing. Vienna, Austria: R Foundation for Statistical Computing; 2014 URL http://www.R-project.org/.

[19] David VMM, MacDonald BA. Seasonal biochemical composition of tissues from *Cucumaria frondosa* collected in the Bay of Fundy, Canada: feeding activity and reproduction. J Mar Biol Assoc UK. 2002; 82: 141–147.

[20] González-Wangüemert M, Domínguez-Godino JA, Aydi M. Profitability of the Mediterranean and NE Atlantic new target sea cucumber species: some repercussions for their fisheries management. Indian J Geo-Mar Sci. 2019; 48: 1715–1719.

[21] Mecheta A, Mezali K. A biometric study to determine the economic and nutritional value of sea cucumbers (Holothuroidea: Echinodermata) collected from Algeria’s shallow water areas. SPC Beche-de-mer Inform Bull. 2019; 39: 65–70.

[22] Hamel JF, Sun J, Gianasi BL, Montgomery EM, Kenchington EL, Burel B. et al Active buoyancy adjustment increases dispersal potential in benthic marine animals. J Anim Ecol. 2019; 88: 820–832. 10.1111/1365-2656.12943 30636040 PMC6850204

[23] Sheehan EV. Motion in the ocean—Paradigm shift in movement ecology requires “sedentary” organisms to be redefined. J Anim Ecol. 2019; 88: 816–819. 10.1111/1365-2656.13006 31168832

[24] Purcell SW, Samyn Y, Conand C. Commercially important sea cucumbers of the world. Rome: FAO; 2012.

[25] Gudimova EN, Gudimov A, Collin P. A study of the biology for fishery in two populations of *Cucumaria frondosa* in the Barents Sea (Russia) and in the Gulf of Maine (USA). In: Heinzeller T, Nebelsick JH, editors. Echinoderms: München, Proceedings of the 11th International Echinoderm Conference. Leriden, Germany: AA Balkema Publishers; 2004. pp 269–275.

[26] So JJ. Assessment of the Biology, Ecology and Gene Flow of the Sea Cucumber Cucumaria frondosa for Management of the Fishery in the Newfoundland and Labrador Region. M.Sc. Thesis, St. John’s: Memorial University. 2009. Available from: https://research.library.mun.ca/view/creator_az/So = 3AJustin_James = 3A = 3A.html

[27] Legault C, Himmelman JH. Relation between escape behaviour of benthic marine invertebrates and the risk of predation. J Exp Ma. Biol Ecol. 1993; 170: 55–74.

[28] Gianasi BL, Verkaik K, Hamel JF, Mercier A. 2015. Novel use of PIT tags in sea cucumbers: promising results with the commercial species *Cucumaria frondosa*. PLoS One 2015; 10: e0127884 10.1371/journal.pone.0127884 26011165 PMC4444348

[29] Brown NA, Wilson DR, Gagnon P. 2019. Plasticity in the antipredator behavior of the orange-footed sea cucumber under shifting hydrodynamic forces. Curr Zool. 2019; 65: 685–695. 10.1093/cz/zoy100 31857815 PMC6911852

[30] Motokawa T. Skin of sea cucumbers: the smart connective tissue that alters mechanical properties in response to external stimuli. J Aero Aqua Bio-Mech. 2019; 8: 2–5.

[31] Neil DM. Ensuring crustacean product quality in the post-harvest phase. J Invert Pathol. 2012; 110: 267–275. 10.1016/j.jip.2012.03.009 22433999

[32] Schram E, Goedhart PW, Molenaar P. Effects of abiotic variables on the survival of discarded bycatches in North Sea pulse-trawl fisheries (No. C040/19). Jmuiden: Wageningen Marine Research 2019 Available from: https://research.wur.nl/en/publications/effects-of-abiotic-variables-on-the-survival-of-discarded-bycatch

[33] Hamel JF, Mercier A. Studies on the reproductive biology of the Atlantic sea cucumber *Cucumaria frondosa*. Gonad morphology and gametogenesis of the sea cucumber *Cucumaria frondosa*. SPC bêche-de-mer Inform Bull. 1996; 8: 22–33.

[34] Hamel JF, Mercier A. Diet and feeding behaviour of the sea cucumber *Cucumaria frondosa* in the St. Lawrence estuary, eastern Canada. Can J Zool. 1998; 76: 1194–1198.

[35] So JJ, Hamel JF, Mercier A. Habitat utilisation, growth and predation of *Cucumaria frondosa*: implications for an emerging sea cucumber fishery. Fisheries Manag Ecol. 2010; 17: 473–484.

[36] Blais M., Galbraith PS, Plourde S, Scarratt M, Devine L, Lehoux C. Chemical and Biological Oceanographic Conditions in the Estuary and Gulf of St. Lawrence during 2017 DFO Can Sci Advis Sec Res Doc. 2019/009. Fisheries and Oceans Canada; 2019.

[37] Starr M., St-Amand L., Berard-Therriault L. État de phytoplancton dans l’estuaire et le golfe du Saint-Laurent en 2001 DFO Can Sci Advis Sec Res Doc. 2002/067. Fisheries and Oceans Canada; 2002.

[38] Mercier A., Hamel JF. Endogenous and exogenous control of gametogenesis and spawning in echinoderms. Adv Mar Biol. 2009; 55: 1–320. 10.1016/S0065-2881(09)55001-8 19595321

[39] Baillon S., Hamel JF, Mercier A. Comparative study of reproductive synchrony at various scales in deep-sea echinoderms. Deep Sea Res Pt I. 2011; 58: 260–272.

[40] Molinet C, Moreno CA, Niklitschek E, Matamala M, Neculman M, Arevalo Nazrala A, et al Reproduction of the sea urchin *Loxechinus albus* across a bathymetric gradient in the Chilean Inland. Sea Rev Biol Mar Oceanog. 2012; 47: 257–272.

[41] Ross DA, Hamel JF, Mercier A. 2013. Bathymetric and interspecific variability in maternal reproductive investment and diet of eurybathic echinoderms. Deep-Sea Res Part II. 2013; 94: 333–342.

[42] Singh R, MacDonald BA, Lawton P, Thomas ML. The reproductive biology of the dendrochirote sea cucumber *Cucumaria frondosa* (Echinodermata: Holothuriodea) using new quantitative methods. Invertebr Reprod Dev. 2001; 40: 125–141.

[43] Hamel JF, Mercier A. Evidence of chemical communication during the gametogenesis of holothuroids. Ecol. 1996; 77: 1600–1616.

[44] Campagna S, Lambert J, Archambault P. Abondance et distribution du concombre de mer (*Cucumaria frondosa*) et prises accidentelles obtenues par dragage entre Matane et Cap-Gaspé (Québec) en 2004 Rapp tech can sci halieut aquat. 2620. Fisheries and Oceans Canada; 2005.

[45] Hamel JF, Mercier A. Early development, settlement, growth, and spatial distribution of the sea cucumber *Cucumaria frondosa* (Echinodermata: Holothuroidea). Can J Fish Aquat Sci. 1996; 53:253–271.

[46] Jordan AJ, 1972. On the ecology and behavior of Cucumaria frondosa (Echinodermata: Holothuroidea) at Lamoine Beach, Maine. Ph.D. Thesis. The University of Maine.1972.

[47] Grant SM. Biological resource assessment of the orange footed sea cucumber (*Cucumaria frondosa*) occurring in the Strait of Belle Isle. Report No. P-169. St. John’s, NF, Canada: Fisheries and Marine Institute Centre for Sustainable Aquatic Resources; 2006.

[48] Feindel S. 2002. Status of the Maine sea cucumber (*Cucumaria frondosa*) fishery Report to the standing legislative committee on marine resources. Maine, USA: Department of Marine Resources; 2002.

[49] Hannah L, Duprey N, Blackburn J, Hand CM, Pearce CM. Growth rate of the California sea cucumber *Parastichopus californicus*: measurement accuracy and relationships between size and weight metrics. N Am J Fish Manage. 2012; 32: 167–176.

[50] Peters-Didier J, Pardo LM, Garrido O, Gallardo CS 2018. Reproductive biology of the commercial sea cucumber *Athyonidium chilensis* (Holothuroidea: Dendrochirotida) in southern Chile. J Mar Biol Association UK. 2018; 98: 311–323.

