# Supplementary material for: Morphometrics and processing yield of Cucumaria frondosa (Holothuroidea) from the St. Lawrence Estuary, Canada
Source: PLoS One. 2021 Jan 22;16(1):e0245238. doi: 10.1371/journal.pone.0245238 (PMC7822298; doi:10.1371/journal.pone.0245238)
Supplement: S1 Table — (DOCX) [file pone.0245238.s003.docx]

**S1-Table. Sampling locations, depths and time of *Cucumaria frondosa* in 2018 on the South shore of the St. Lawrence Estuary, QC, Canada**.

| **ID** | **Fishing**  **method** | **Fishing**  **area** | **Depth**  **(m)** | **Latitude** | **Longitude** | **Collection date**  **Month, Day** | **Dissection date**  **Month, Day** |
| --- | --- | --- | --- | --- | --- | --- | --- |
| M-A11 | Diving  Scientific | A | 10.7 | 49.15 | -66.45 | May, 28 | June, 26–27 |
| J-A09 | Diving  Scientific | A | 8.5 | 49.16 | -66.46 | July, 17 | July, 23–24 |
| S-AA13 | Diving  Commercial | AA | 13.4 | 48.93 | -67.25 | September, 10 | September, 11 |
| O-B16 | Dredging | B | 15.9 | 49.23 | -65.94 | October, 9 | October, 9–10 |
| O-B26 | Dredging | B | 25.9 | 49.23 | -65.85 | October, 9 | October, 9–10 |
| O-B41 | Dredging | B | 41.1 | 49.26 | -65.52 | October, 8 | October, 8–9 |
| O-B47 | Dredging | B | 46.7 | 49.26 | -65.42 | October, 8 | October, 8–9 |
| O-C29 | Dredging | C | 28.7 | 48.90 | -64.22 | October, 4 | October, 4–5 |
| O-C35 | Dredging | C | 35.0 | 49.01 | -64.38 | October, 4 | October, 4–5 |
| O-C45 | Dredging | C | 45.1 | 49.02 | -64.39 | October, 6 | October, 6–7 |
