# Supplementary material for: Morphometrics and processing yield of Cucumaria frondosa (Holothuroidea) from the St. Lawrence Estuary, Canada
Source: PLoS One. 2021 Jan 22;16(1):e0245238. doi: 10.1371/journal.pone.0245238 (PMC7822298; doi:10.1371/journal.pone.0245238)
Supplement: S2 Table — Samples were collected by diving. (DOCX) [file pone.0245238.s004.docx]

**S2-Table. Two-way ANOVA table with sampling time and sex as main factors for whole, gutted, and processed body mass and length. Samples were collected by diving.**

| Variable | Time | | | Sex | | | Time X Sex | | |
| --- | --- | --- | --- | --- | --- | --- | --- | --- | --- |
|  | F | df | p | F | df | p | F | df | p |
| Whole body |  |  |  |  |  |  |  |  |  |
| Mass | 34.32 | 2 | ≤ 0.001 | 19.30 | 1 | ≤ 0.001 | 6.35 | 2 | 0.002 |
| Length | 8.76 | 2 | ≤ 0.001 | 10.11 | 1 | 0.002 | 5.64 | 2 | 0.004 |
| Gutted body |  |  |  |  |  |  |  |  |  |
| Mass | 3.94 | 2 | 0.02 | 27.97 | 1 | ≤ 0.001 | 2.41 | 2 | 0.09 |
| Length | 0.44 | 2 | 0.65 | 11.88 | 1 | ≤ 0.001 | 0.03 | 2 | 0.97 |
| Processed body |  |  |  |  |  |  |  |  |  |
| Mass | 2.73 | 2 | 0.07 | 35.59 | 1 | ≤ 0.001 | 1.93 | 2 | 0.15 |
| Length | 1.78 | 2 | 0.17 | 17.92 | 1 | ≤ 0.001 | 0.64 | 2 | 0.53 |
