# Supplementary material for: Morphometrics and processing yield of Cucumaria frondosa (Holothuroidea) from the St. Lawrence Estuary, Canada
Source: PLoS One. 2021 Jan 22;16(1):e0245238. doi: 10.1371/journal.pone.0245238 (PMC7822298; doi:10.1371/journal.pone.0245238)
Supplement: S3 Table — Samples were collected by dredging. (DOCX) [file pone.0245238.s005.docx]

**S3-Table. Two-way ANOVA table with sampling site and sex as main factors for whole, gutted, and processed body mass and length. Samples were collected by dredging.**

| Variable | Site | | | Sex | | | Site X Sex | | |
| --- | --- | --- | --- | --- | --- | --- | --- | --- | --- |
|  | F | df | p | F | df | p | F | df | p |
| Whole body |  |  |  |  |  |  |  |  |  |
| Mass | 24.94 | 6 | ≤ 0.001 | 0.59 | 1 | 0.44 | 0.74 | 6 | 0.61 |
| Length | 36.24 | 6 | ≤ 0.001 | 0.01 | 1 | 0.91 | 0.34 | 6 | 0.91 |
| Gutted body |  |  |  |  |  |  |  |  |  |
| Mass | 23.58 | 6 | ≤ 0.001 | 3.25 | 1 | 0.07 | 1.17 | 6 | 0.33 |
| Length | 7.45 | 6 | ≤ 0.001 | 0.35 | 1 | 0.56 | 0.63 | 6 | 0.71 |
| Processed body |  |  |  |  |  |  |  |  |  |
| Mass | 22.53 | 6 | ≤ 0.001 | 2.62 | 1 | 0.11 | 1.43 | 6 | 0.20 |
| Length | 8.27 | 6 | ≤ 0.001 | 4.33 | 1 | 0.04 | 0.67 | 6 | 0.67 |
